# δ-Opioid Receptors, microRNAs, and Neuroinflammation in Cerebral Ischemia/Hypoxia

**DOI:** 10.3389/fimmu.2020.00421

**Published:** 2020-03-25

**Authors:** Yi-Meng Chen, Xiao-Zhou He, Shu-Ming Wang, Ying Xia

**Affiliations:** ^1^Department of Urology, The Third Affiliated Hospital of Soochow University, Changzhou, China; ^2^Department of Anesthesiology, University of Connecticut, Mansfield, CT, United States; ^3^Shanghai Key Laboratory of Acupuncture Mechanism and Acupoint Function, Fudan University, Shanghai, China

**Keywords:** brain injury, hypoxia, ischemia, microRNAs, δ-opioid receptor (DOR), neuroinflammatory response

## Abstract

Hypoxia and ischemia are the main underlying pathogenesis of stroke and other neurological disorders. Cerebral hypoxia and/or ischemia (e.g., stroke) can lead to neuronal injury/death and eventually cause serious neurological disorders or even death in the patients. Despite knowing these serious consequences, there are limited neuroprotective strategies against hypoxic and ischemic insults in clinical settings. Recent studies indicate that microRNAs (miRNAs) are of great importance in regulating cerebral responses to hypoxic/ischemic stress in addition to the neuroprotective effect of the δ-opioid receptor (DOR). Moreover, new discovery shows that DOR can regulate miRNA expression and inhibit inflammatory responses to hypoxia/ischemia. We, therefore, summarize available data in current literature regarding the role of DOR and miRNAs in regulating the neuroinflammatory responses in this article. In particular, we focus on microglia activation, cytokine production, and the relevant signaling pathways triggered by cerebral hypoxia/ischemia. The intent of this review article is to provide a novel clue for developing new strategies against neuroinflammatory injury resulting from cerebral hypoxia/ischemia.

## Introduction

Neurons in the mammalian central nervous system are extremely vulnerable to deprivation of oxygen and blood supply. Once the neurons are deprived of oxygen or blood supply, many pathological events are triggered including inflammatory changes in the brain. In addition, local accumulation of metabolic wastes also leads to local/regional tissue dysfunction and/or damage (1). Moreover, the reestablishment of blood flow and/or oxygen supply further enlarges the area of cell death and/or tissue damage secondary due to the activation of immune responses and cell death programs (2). Although the past studies have identified numerous events/molecules that are critical determinants of neural survival/death under hypoxic/ischemic conditions, there is still a paucity of neuroprotective strategies against hypoxic/ischemic insults in the clinical settings. Therefore, there is an urgent need to advance our understanding of hypoxic/ischemic process and explore various novel therapies against the cerebral injury caused by hypoxia/ischemia.

Thus far, microRNAs (miRNAs) and DOR have been identified as key factors in regulating neuroinflammatory and other biological processes under cerebral hypoxia and ischemia. MicroRNAs are short RNA molecules (a class of ~21- to 25-ribonucleotide single-strand non-coding RNA molecules) and can be found in all eukaryote cells (3). They bind to target messenger RNAs (mRNAs) through base pairing with the 3′ untranslated regions (3′-UTRs), modulating direct cleavage and/or translational repression of target mRNAs (4). Up to now, more than 2,000 human miRNAs have been identified (5). These miRNAs are found to play important roles in a wide spectrum of processes under both physiological and pathological conditions, for example, hypoxia and ischemia. The spatiotemporal properties of miRNA expression provide complex regulatory networks in the mammalian cells. Thus, a better understanding of miRNAs and their roles in repression of target mRNA and gene silencing is warranted to unveil the potential therapeutics against hypoxic/ischemic injury by targeting specific miRNA molecules in the brain.

Opioid receptors belong to the large family of seven transmembrane G protein–coupled receptors. There are three major types of opioid receptors, the μ-, κ-, and δ-opioid receptors (known as MOR, KOR, and DOR) (6). Although there exists only one DOR gene, two DOR subtypes, namely, DOR1 and DOR2, are identified according to their pharmacological attributes (7). High levels of endogenous DOR mRNA are expressed in the brain (especially in cortex and striatum) and dorsal root ganglion (6, 8–10). Our previous studies have shown that DOR activation and/or overexpression induce a protective effect against neuronal injury in hypoxic/ischemic conditions (11, 12). Such observations were made through serial studies on primary cultured neurons under hypoxia (13–15), brain slices in oxygen–glucose deprivation (OGD) (16–20), and *in vivo* brain exposed to cerebral hypoxia/ischemia (21–24). Our observations on DOR neuroprotective effect have been confirmed by other independent laboratories (25–32). δ-opioid receptor neuroprotection is mediated by several important pathways, including an increase in cellular antioxidant activity and inhibition of cell death signaling. Moreover, there is growing evidence suggesting that the DOR neuroprotection against hypoxic/ischemic injury may be achieved by modulating miRNAs because DOR regulates miRNA expression in different organs such as brain, kidney, heart, and liver in hypoxia (33–36). Therefore, it is possible to protect organs against hypoxic/ischemic injury by targeting specific miRNA molecules directly or indirectly through DOR signaling.

In this article, we reviewed the effects of DOR activation on miRNAs and neuroinflammatory responses to hypoxic/ischemic insults. First, we discussed the effect of hypoxia/ischemia on the expression of cerebral miRNAs. Second, we summarized the miRNA-mediated neuroinflammatory events under hypoxic/ischemic conditions. Third, we indicated various miRNAs involved in microglia activation, cytokine production, and cell signaling under hypoxia and ischemia. Fourth, we discussed the effect of DOR activation on the miRNA expression. Finally, we briefly commented on the potential use of circulating miRNAs as biomarkers and possible targets for clinical treatment against hypoxic/ischemic injury.

## Effects of Hypoxia/Ischemia on Cerebral miRNA Expression

The biogenesis of miRNA in mammalian cells required multistep process that begins with transcription of the primary miRNAs (pri-miRNAs) by RNA polymerase II in the nucleus. MicroRNA genes are transcribed either from introns of protein-coding genes or by intergenic miRNAs under the control of their own promoters (37). Primary miRNAs are cleaved by microprocessors including DROSHA and DiGeorge syndrome critical region 8 (DGCR8), to produce the ~60- to 70-nucleotide stem-loop precursor miRNAs (pre-miRNAs). The pre-miRNAs are then exported to the cytoplasm via exportin-5 and further processed by Dicer. One strand of the mature miRNA is loaded into the RNA-induced silencing complex (RISC), whereas the remaining strand is released and degraded. Mature miRNA guides RISC to target transcripts by sequence complementary binding and mediates gene suppression (38). Current literature suggests that hypoxia/ischemia can regulate miRNA expression at various steps throughout its biogenesis pathway. For instance, transcriptional activities of miRNA genes can be affected by epigenetic modifications (e.g., DNA methylation and histone modification) and/or binding of different transcriptional factors [e.g., HIF, nuclear factor (NF-κB), and p53] that are involved in various biological and inflammatory responses. Hypoxia/ischemic condition also affects the expression of some enzymes, e.g., Drosha, Dicer, and AGO2, which participate in the regulation of pri-miRNA processing and the maturation of various miRNAs. Finally, miRNA–RISC complex configurations are modulated under hypoxic/ischemic conditions (39, 40).

Current research has shown that miRNAs play an important role in response to the hypoxic/ischemic insults to the brain, a hypoxia/ischemia–sensitive organ (34, 39–42). Indeed, cerebral hypoxic/ischemic stress regulates miRNA expression and significantly affects neuronal functions and survival (Table 1). In addition, clinical studies also showed that stroke patients had a dysregulation in global miRNA profiles several months after the original hypoxic/ischemic insults (76, 77). It is reported that mild/moderate hypoxic/ischemic stress may induce cell proliferation, migration, and angiogenesis (78, 79), whereas a severe/prolonged hypoxia/ischemic stress causes apoptosis and necrosis. The differential cellular signaling is partially mediated by the miRNA-induced repression of gene expression (62, 80).

**Table 1:** Hypoxia/ischemia–induced miRNA changes in the brain with defined targets.

**Hypoxia/ischemia regulated miRNAs**	**Species**	**Target genes**	**Function**	**References**
**UP-REGULATED BY HYPOXIA/ISCHEMIA**				
miR-1	Mouse	HSP70	Induce DNA fragmentation and neuronal cell apoptosis	(43)
miR-27a	Rat	LAMP2	Influence lysosomal clearance and autophagy	(44)
miR-29a	Rat	PUMA	Maintain mitochondrial function	(45)
miR-106b-5p	Human, rat	MCL1	Promote apoptosis and oxidative stress	(46)
miR-130a	Rat	HOXA5	Regulate cerebral ischemia–induced blood–brain barrier permeability	(47)
miR-200b	Rat	KLF4	Regulate microglial M1/M2 polarization	(48)
miR-200c	Mouse	RELN	Induce oxidative injury and neuronal death	(49)
miR-210	Rat	GR	Promote hypoxia/ischemia–induced neuronal death	(50)
	Mouse	NP1	Glutamate-mediated excitotoxicity to cortical neurons	(51)
	Mouse	ISCU1/2	Control mitochondrial metabolism	(52)
miR-215	Human	KDM1B	Angiogenesis, glucose metabolism, and chondroitin sulfate modification	(53)
miR-365	Rat	PAX6	Modulate astrocyte-to-neuron conversion	(54)
miR-497	Mouse	BCL2	Proapoptosis and ischemic neuronal death	(55)
miR-3473b	Mouse	SOCS3	Promote neuroinflammation	(56)
**DOWN-REGULATED BY HYPOXIA/ISCHEMIA**				
miR-7	Mouse	HERP2	Modulate astrocytic inflammatory responses	(57)
	Rat	SNCA	Improve motor and cognitive function	(58)
miR-9	Mouse	BCL2L11	Antineuronal apoptosis	(59)
miR-21	Mouse	PDCD4	Modulate oxygen–glucose deprivation and apoptotic cell death	(60)
	Rat	FasL	Modulate neuronal apoptosis and microglia activation	(61)
miR-23b/27b	Mouse	APAF1	Antineuronal apoptosis	(62)
miR-29b	Human, mouse	AQP4	Edema and blood–brain barrier disruption	(63)
miR-122	Human	G6PC3, ALDOA, CS	Regulate glucose and energy metabolism	(64)
miR-124	Human	TEAD1, MAPK14, SERP1	Counteract prosurvival stress responses in glioblastoma	(65)
miR-125b	Rat	TP53INP1	Inhibit neuroinflammation and apoptosis	(66)
miR-135a/199a-5p	Human	FLAP	Increase leukotriene formation	(67)
miR-139-5p	Rat	HGTD-P	Inhibit neuronal apoptosis	(68)
miR-181c	Rat	TLR4	Modulate NF-κB activation and neuroinflammation	(69)
miR-374a	Human	ACVR2B	Modulate immune response	(70)
miR-377	Rat	VEGF, EGR2	Modulate cerebral inflammation	(71)
miR-424	Human, mouse	CDC25A, CCND1, CDK6	Neuronal apoptosis and microglia activation	(72)
miR-592	Mouse	NTR	Antiapoptotic cell death	(73)
let-7c-5p	Human, mouse	Caspase 3	Inhibit microglia activation	(74)
let-7i	Human	CD86, CXCL8, HMGB1	Regulate leukocyte activation, recruitment, and proliferation	(75)

*These changes are summarized from the existing literature. As we indicated in the text, their changes in response to hypoxia and/or ischemia may vary, depending on the duration and severity of stress, types of tissues, experimental objects, and so on*.

*HSP70, heat shock protein-70; LAMP2, lysosomal associated membrane protein 2; PUMA, BCL2 binding component 3; MCL1, myeloid cell leukemia-1; HOXA5, homeobox A5; KLF4, Krüppel-like factor 4; RELN, reelin; GR, glucocorticoid receptor; NP1, neuronal pentraxin 1; ISCU1/2, iron–sulfur cluster assembly enzyme 1/2; KDM1B, lysine demethylase 1B; PAX6, paired box 6; BCL2, BCL2 apoptosis regulator; SOCS3, suppressor of cytokine signaling 3; HERP2, HERPUD family member 2; SNCA, α-synuclein; BCL2L11, BCL2 like 11; PDCD4, programmed cell death 4; FasL, Fas ligand; APAF1, apoptotic protease activating factor-1; AQP4, aquaporin-4; G6PC3, glucose-6-phosphatase catalytic subunit 3; ALDOA, aldolase, fructose–bisphosphate A; CS, citrate synthase; TEAD1, TEA domain 1; MAPK14, MAP kinase 14; SERP1, stress-associated endoplasmic reticulum protein; TP53INP1, tumor protein p53 inducible nuclear protein 1; FLAP, 5-lipoxygenase activating protein; HGTD-P, human growth transformation dependent protein; TLR4, Toll-like receptor 4; ACVR2B, activin-A receptor type IIb; VEGF, vascular endothelial growth factor; EGR2, early growth response gene 2; CDC25A, cell division cycle 25A; CCND1, cyclin D1; CDK6, cyclin dependent kinase 6; NTR, neurotrophin receptor p75; CXCL8, C-X-C motif chemokine ligand 8; HMGB1, high-mobility group box 1*.

Hypoxia-inducible factor 1 (HIF-1) lies at the epicenter of the complex regulatory network that involves hundreds of protein-coding genes and directly regulates the expression of certain miRNAs via its transcriptional factor activity in the brain. It is a key transcriptional factor for cerebral hypoxic/ischemic responses (81, 82). MiR-210 was one of the first hypoxia-sensitive miRNAs discovered as a direct transcriptional target of HIF (83). Upon hypoxic exposure, miR-210 transcription is dynamically induced through HIF-1α interaction with the hypoxia-response element (HRE) located within its promoter region (40, 52). Evidence indicates that HIF-1 may also contribute to the transcriptional activation of other hypoxia-sensitive miRNAs (e.g., miR-26, miR-181, and miR-26) through direct binding to HREs in their respective promoter regions (83, 84). The regulation has been confirmed by experiments including luciferase-based promoter-reporter assays and chromatin immunoprecipitation (ChIP) assays (83).

Several studies have attempted to summarize the complex hypoxia/ischemia–induced miRNA alternations in the brain and found that the hypoxic/ischemic miRNA changes are highly variable following the different durations of hypoxia/ischemia, the level of oxygen concentration, and the types of cell/animal models. Different miRNAs exhibit diverse responses to different durations of hypoxic/ischemic insults in the same brain region. Our previous study examined the effects of hypoxia on miRNA expression in the rat cortex. The expression of miR-29b, miR-101b, miR-298, miR-324-3p, miR-347, and miR-466b were significantly down-regulated following 1-day exposure to hypoxia. However, some other miRNAs were more tolerant to hypoxic insult. For instance, a down-regulated expression of miR-31 and miR-186 was observed only following a continuous 5-day hypoxia exposure. The expression of miR-29a, let-7f, and miR-511 remain unchanged until a continuous 10-day exposure of hypoxia (34). These findings suggest that cortical miRNAs respond differently to hypoxic stress, depending on the durations of exposure.

**Figure 1:**
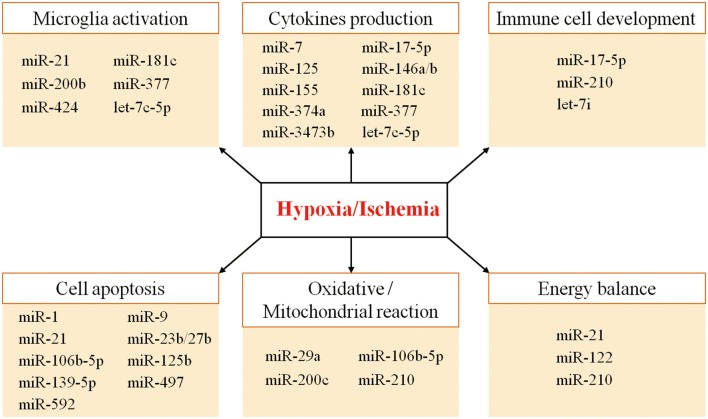
The diverse functions of the miRNAs involved in hypoxic/ischemic responses.

Thus, the combinatorial and coordinated regulation of miRNAs forms a complex network to regulate target genes in response to hypoxic/ischemic insult. As illustrated in Figure 1, many miRNAs regulate neuroinflammation events such as microglia activation, cytokine production, and immune cell development, as well as other biological processes such as apoptosis, oxidative/mitochondrial reaction, and energy balance in responding to cerebral hypoxia/ischemia (Figure 1). For instance, the hypoxia-sensitive miR-210 is potently induced by hypoxia via an HIF-1–dependent manner and in turn stabilizes HIF-1 by targeting glycerol-3-dehydrogenase-like 1, forming a positive feedback regulatory loop (85). Table 1 summarizes miRNA changes in cerebral hypoxia/ischemia with defined target genes based on both animal and clinical studies.

## The miRNA-Mediated Neuroinflammatory Events Under Hypoxia/Ischemia

Hypoxic and/or ischemic injuries are well-documented entities in the pathogenesis of cerebrovascular diseases such as stroke, and neurodegenerative diseases, including Alzheimer's disease (AD) and Parkinson's disease (PD). The effects of hypoxia/ischemia on miRNA expression in the brain have been widely investigated in different animal models and clinical settings. Khoshnam et al. (86) reviewed the interplay of miRNAs in the inflammatory processes following ischemic stroke.

Cerebral hypoxia/ischemia greatly affects key inflammatory transcription factors including NF-κB (87). It also induces the synthesis and release of inflammatory mediators, enzymes, and cytokines (87, 88). On the other side, hypoxia/ischemia can greatly alter miRNA expression. A brief summary of hypoxia/ischemia–sensitive miRNAs and their targeting genes involved in neuronal inflammatory and immune responses is shown in Table 1.

MicroRNAs regulate inflammatory response by affecting microglia activation in cerebral hypoxia/ischemia. A significant reduction of miR-424 expression was discovered in circulating lymphocytes of patients with ischemic stroke (72). Similar findings are also observed in mouse plasma and brain tissues after ischemia. In contrast, miR-424 overexpression caused G1 phase cell-cycle arrest by translational suppression of key activators of G1/S transition (e.g., CDC25A, cyclin D1, and CDK6) in microglia cells, thus attenuating ischemic brain injury by inhibiting neuronal apoptosis and microglia activation (72). In an animal model of middle cerebral artery occlusion (MCAO), there is an elevation of miR-200b expression after brain ischemia. MiR-200b up-regulation was able to induce proneuroinflammatory cytokine production and microglia M1 polarization via directly targeting KLF4 (48). Similarly, let-7c-5p was significantly altered in the plasma of patients with ischemic stroke and in MCAO mice (74). The ischemic induction of proinflammatory mediators [e.g., inducible nitric oxide synthase (iNOS), cyclooxygenase 2 (COX-2), tumor necrosis factor α (TNF-α), and interleukin 6 (IL-6)] in the ischemic cortex were attenuated by the overexpression of let-7c-5p (74). The neuroprotective effect is likely achieved by the direct regulation of let-7c-5p on caspase 3 levels, which is an important regulator of microglia M1/M2 polarization (89–91). MiR-377 plays a proinflammatory role following ischemic stroke by modulating the anti-inflammation effect of EGR2 and the proangiogenesis effect of vascular endothelial growth factor (VEGF) (71). Fan et al. (71) found that the level of miR-377 decreased in the rat brain after MCAO and cultured microglia cells exposed to OGD. Furthermore, they found that miR-377 knockdown could attenuate microglia activation and the release of proinflammatory cytokines after OGD. In addition, miR-21 can induce neuronal protection via suppression of FasL and inhibition of microglia activation (61), whereas OGD down-regulates miR-21 expression in rat primary microglial cells, suggesting an up-regulation of miR-21 may induce a protective effect against ischemic injury.

Hypoxia/ischemia–sensitive miRNAs can alter the production of inflammatory cytokines through direct or indirect pathways. For instance, miRNA-181c suppresses the expression of TNF-α, a key inflammatory cytokine, by binding to the 3′-UTR (92). Toll-like receptor 4 (TLR4) was confirmed to be another direct target of miR-181c. Down-regulation of miR-181c expression in primary microglia under hypoxia promoted TLR4 expression and NF-κB activation, increasing the downstream production of proinflammatory mediators (69). MiR-125b was also found to activate the NF-κB signals by targeting a negative NF-κB regulator, for example, the TNF-α-induced protein 3. On the other hand, miR-125b is a direct NF-κB transcriptional target. Thus, there is a positive self-regulatory loop of miR-125–NF-κB for strengthening and prolonging NF-κB activity (93).

Ischemia–reperfusion injury resulted in a reduction of miR-125b in rat brain and increase in protein levels of TP53INP1, p53, cytokines IL-1β, and TNF-α (66). Two hypoxia-related miRNAs, miR-146a and miR-155, are found to play an important role in NF-κB–mediated inflammatory responses following hypoxia (40, 42). Tumor necrosis factor receptor–associated factor 6, IL-1 receptor–associated kinase 1, and TLR4 are direct target genes of miR-146a/b (94–96). Hypoxia can promote miR-146a expression, thus down-regulating expression of proinflammation targets (97). In rat ischemic model, the brain and circulation miR-155 was down-regulated (98). Reducing miR-155 facilitates the downstream proinflammatory signaling via multiple targets including inositol phosphatase SHIP1, MyD88, and SOCS1 required for TLR/IL-1R signaling (99–102). In the endotoxin mouse model, Alexander et al. (103) further confirmed the importance of exosome-delivered miR-155 and miR-146a in regulating the immune responses; for example, miR-146a reduced, whereas miR-155 enhanced the inflammatory responses. There are several examples for the miRNA-modulated production of inflammatory cytokines, including the miR-3473b-SOCS3-iNOS/COX-2/TNF-α/IL-6 axis in stroke pathology (56), miR-374a-ACVR2B-IL-6/IL-8 axis in the infants with hypoxic/ischemic encephalopathy (70), and endoplasmic reticulum stress-related miR-7-HERP2-TNF-α/IL-1β axis in mouse astrocytes exposed to OGD insult (57).

Various studies have demonstrated that miR-210 serves as a key mediator of hypoxia/ischemia–dependent responses. Thus far, a complex spectrum of target genes for miR-210 has been identified. These genes are involved in angiogenesis, cell proliferation, mitochondrial metabolism, inflammatory response, and so on (52, 104–107). MiR-210, through HIF-1α-dependent pathway, can modulate T-cell differentiation in hypoxia, limit inflammatory cytokine production, and decrease the severity of disease (108). MiR-210 also mediates immunosuppression and immune escape under hypoxic conditions in cancer cells (109, 110). Elevated miR-210 in oxygen-deprived regions of tumors decreases cell susceptibility to lysis by antigen-specific cytotoxic T lymphocytes (109). In addition, miR-210 was shown to enhance myeloid-derived suppressor cell–mediated T-cell suppression by targeting Arg1, CXCL12, and IL-16 expression, thus facilitating tumor growth by increasing arginase activity and nitric oxide production (110). Interestingly, germline deletion of miR-210 in mice resulted in the development of autoantibodies, whereas overexpressing miR-210 exhibited impaired class-switched antibody responses, expanding the immune regulatory function of miR-210 to B cell activation and autoantibody production (111). Taken together, these findings underscored a key regulatory role for hypoxia-induced miR-210 in immune cell development. Apart from well-studied miR-210, decreased expression of let-7i and miR-17-5p were discovered in patients with acute ischemic stroke and in animal post–hypoxia/ischemia models. Let-7i regulates several signals involved in leukocyte activation, recruitment, and proliferation involving of CD86 signaling in T helper cells, high-mobility group box 1 (HMGB1) signaling, and CXCL8 signaling (75). MiR-17-5p regulates NLRP3 inflammasome activation, caspase 1 cleavage, and IL-1β production in the rat model of brain damage (112).

The NF-κB signaling is a key factor in inflammatory responses and functions to modulate several hypoxic/ischemic–sensitive miRNAs. This phenomenon has been observed in both acute and chronic hypoxic conditions (113, 114). Systematic screening approach was applied in identifying several NF-κB–regulated miRNAs, including miR-146a, miR-155, miR-21, miR-130, miR-210, and so on (94, 115–117). Researchers have discovered the NF-κB binding sites located at the promoter regions of miR-21, miR-130, and miR-210. Using ChIP and luciferase reporter assays, direct interactions between NF-κB and promoters of miR-21, miR-130, and miR-210 have been confirmed (116, 117).

In conclusion, multiple miRNAs and transcriptional factors form positive or negative feedback regulatory loops that actively participate in hypoxia/ischemia–induced neuroinflammatory responses.

## δ-Opioid Receptor–Induced Alterations in miRNA Expression in the Brain Under Hypoxic Condition

Our recent studies and those of others have provided a strong evidence supporting DOR-mediated neuroprotection in the brain, especially in the cortex (7). An important aspect of the DOR-mediated neuroprotection is its ability in maintaining cellular ionic homeostasis. Specially, DOR activation attenuates Na^+^ influx through the membrane, reduces the increase in intracellular Ca^2+^, and decreases the excessive leakage of intracellular K^+^ (12, 16–20). δ-opioid receptor activation reduces hypoxic/ischemic disruption of ionic homeostasis by inhibiting the Na^+^ channels and *N*-methyl-d-aspartate receptors (19) and activating a PKC-dependent signaling pathway (16, 20). Another aspect of DOR-mediated neuroprotection is the reduction of glutamate-induced excitotoxic neuronal injury by enhancing antioxidant ability and inhibiting caspase signaling (15, 21, 118, 119). δ-opioid receptor activation can also inhibit the production of inflammatory cytokines in hypoxic/ischemic conditions. In a rat model, DOR activation improved rat survival, which is associated with a significant decrease in release of proinflammatory molecules (e.g., TNF-α, IL-1β, and HMGB1) (120). δ-opioid receptor activation suppressed TNF-α expression and retinal ischemia-induced cell death (121). Our studies discovered that DOR activation has an inhibitory effect on hypoxia-induced increase in TNF-α in cultured rat astrocytes (122). Moreover, DOR plays an important role in neuroprotection against hypoxic/ischemic stress by regulating the expression of inflammatory and anti-inflammatory cytokines in the neonatal brain (123). The underlying mechanism is partially mediated by Nrf-2/HO-1/NQO-1 signaling (123) and, at least partially, involved in DOR-mediated miRNA regulation.

**Figure 2:**
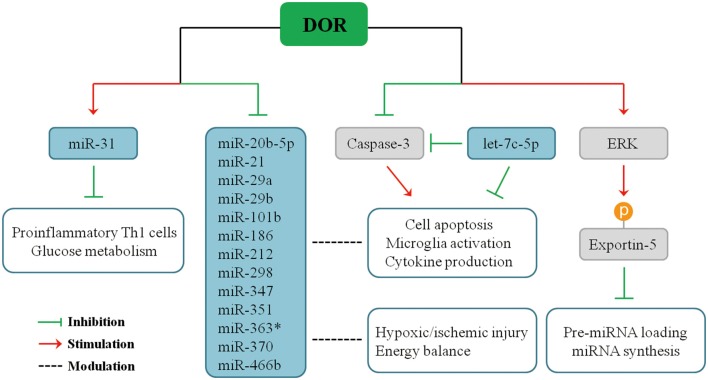
Influences of DOR activation on miRNA expression in prolonged hypoxia.

Recent studies demonstrated that DOR activation modulates the expression of miRNAs in multiple organs in response to hypoxic stress. Our serial studies demonstrated that DOR activation has a positive or negative impact on different miRNAs in the brain, kidney, heart, and liver in hypoxia. Using microarray and quantitative real-time polymerase chain reaction (PCR) analysis to measure miRNA expression and applying UFP-512, a potent and specific DOR agonist, to activate DOR in Sprague–Dawley rats (33–36), we found that some miRNAs significantly change their expression upon DOR activation in the brain under normoxic condition, and such modulation becomes more profound in hypoxic/ischemic conditions, especially after a prolonged period. For instance, DOR activation did not alter the brain miR-31 expression under normoxic condition, but it led to a 50% increase in miR-31 levels after 1-day hypoxic exposure, suggesting that DOR activation up-regulates miR-31 in hypoxia (34), thus inhibiting proinflammatory T_H_1 cells and cell glucose metabolism (124, 125). In the same animal model, DOR activation reduced the levels of miR-347 and miR-466b in the cortex after prolonged hypoxia as compared to DOR activation in normoxic animals (34). Moreover, 5-day hypoxia had no significant effect on cortical miR-21 and miR-370 expression. However, the combination of hypoxia and DOR activation produced a dramatic 70% suppression in both miR-21 and miR-370 levels. Moreover, cortical miR-21 and miR-370 expression remained unchanged when DOR agonist was applied under normoxic conditions, and the regulatory effect of DOR was shown when the animals were exposed to prolonged hypoxia (34). Similarly, many other cortical miRNAs had a sensitive response to DOR activation and changed their expression in hypoxic condition, including miR-20-5p, miR-29a, miR-29b, miR-101b, miR-186, miR-212, miR-298, miR-351, and miR-363^*^ (34). The effects of DOR activation on miRNA expression after pronged hypoxia are summarized in Figure 2.

Therefore, DOR activation can affect miRNA-mediated neuroinflammatory responses under hypoxic/ischemic conditions through direct or indirect pathways. The expression of miR-21, miR-29a, and miR-29b are directly regulated upon DOR activation and thus affecting cellular events mediated by target genes. On the other side, DOR activation modulates some key molecules and transcriptional factors and affects miRNA biogenesis or cellular inflammatory responses, eventually affecting neuronal survival/death. We have found that DOR activation up-regulates extracellular-signal-regulated kinase (ERK) activity, and ERK signaling mediates DOR neuroprotection (14). ERK is found to suppress pre-miRNA export from the nucleus to cytoplasm through phosphorylation of exportin-5, resulting in a global reduction of pre-miRNA loading and miRNA synthesis in cancer cells (126, 127). The similar regulatory mechanism may exist in the brain tissue. Besides ERK signaling, DOR activation also reduces caspase 3 expression (21). Mounting evidence suggests that caspase 3 is an important regulator of microglia activation and inflammation-mediated neurotoxicity independently from its apoptotic activity. Indeed, activated caspase 3 in microglia was found in several neurodegenerative disease models including PD and AD (91), partially accounting for neuroinflammation in the neurodegenerative brains. Caspase inhibitors can hinder microglia activation and consequently reduce neuroinflammation (91, 128, 129). Interestingly, caspase 3 is a direct target of let-7c-5p (74), suggesting a potential regulatory network among DOR, let-7c-5p, caspase signaling, and microglia-mediated neuroinflammation.

## Potential Clinical Applications and Therapeutic Targets

MicroRNAs have been implicated as an important player in response to brain hypoxia/ischemia. Emerging evidence shows that multiple hypoxia/ischemia–sensitive miRNAs may serve as potential clinical biomarkers for brain injury and the progression of neurological disorders (130). In addition, certain circulatory miRNAs are proved to correlate with their changes in the brain with pathological alterations (131). These specific circulatory miRNA(s) may serve as a biomarker(s) for neuropathological changes and the prognosis. For example, a positive correlation between blood and brain levels of miR-210, a master and pleiotropic miRNA sensitive to hypoxia, was reported in patients with ischemic stroke and ischemic mouse models. Moreover, the levels of miR-210 in stroke patients with good outcomes were significantly higher than those in patients with poor outcomes (132). Tiedt et al. (133) identified circulating miR-125a-5p, miR-125b-5p, and miR-143-3p as potential biomarkers for acute ischemic stroke by RNA sequencing followed by real-time PCR validation. Liu et al. (98) also performed brain and whole-blood miRNA expression profiles in rat models with different neurological disorders and identified a set of blood miRNAs (e.g., miR-298, miR-155, and miR-362-3p) that are correlated with different disease statuses. All these studies strongly suggest that certain blood miRNAs can serve as biomarkers for hypoxia/ischemia–relevant neurological diseases. Further studies on miRNAs that respond to hypoxia/ischemia and their regulatory mechanisms may lead to major improvements in diagnostic medicine and disease prognosis.

A large number of mediators and intracellular signaling are actively participating in neuroinflammatory responses to hypoxia/ischemia. Targeting selected miRNAs that have the capacity to modulate these inflammatory mediators and signaling may be potentially used to develop new therapies. For instance, IL-10 is an anti-inflammatory molecule participating in tissue repair and cytoprotective effects in ischemic brain tissue (134). Interleukin 10 can be directly regulated by several miRNAs, for example, miR-106a, miR-4661, miR-98, miR-27, let-7, and miR-1423 post-transcriptionally (135). Moreover, miR-21 can indirectly regulate IL-10 via down-regulation of the IL-10 inhibitor PDCD4 [Table 1; (136)]. An interaction between miRNAs and IL-10 has been implicated to play a vital role in inflammatory and autoimmune diseases (137, 138). Moreover, inhibition of miR-155 can trigger IL-10–mediated anti-inflammatory responses, resulting in improved post-stroke recovery (139). Furthermore, modulating miR-210 and miR-107 can result in VEGF-associated post-stroke angiogenesis and neurogenesis, suggesting that miR-210 and miR-107 may serve as a potential strategy for stroke treatment (140, 141).

Because DOR is neuroprotective and has a broad influence on miRNA expression in hypoxia, it is likely to develop new protective strategies against hypoxic/ischemic brain injury by directly or indirectly targeting certain miRNAs. There is a high level of endogenous DOR expression in the cortex, striatum, temporal lobe, putamen, and caudate nucleus (6, 8–10, 142). All these brain regions are commonly attacked by stroke and other neurodegenerative diseases. A short-term attack, for example, acute hypoxia, may up-regulate DOR expression (7), but prolonged hypoxia largely reduced the level of DOR in the brain (7, 11, 14). An increase in the DOR expression and/or activity may greatly render these brain regions more tolerant to hypoxic/ischemic insults. Unfortunately, this is a difficult, if not impossible, strategy for the patients at present. Alternatively, applying specific DOR agonist, through miRNA- and non–miRNA-mediated neuroprotection, may be a more reliable clinical approach. However, there are still some limitations in the clinical application of DOR agonists because of opioid addiction/tolerance and other issues such as low specificity of opioid ligands (6, 143). Also, chronic opioid administration has an inhibitory effect on immune ability, for example, immunosuppression, including antibody production, natural killer cell activity, cytokine expression, and phagocytic activity (144). More in-depth research on the molecular mechanisms of DOR-mediated miRNA regulation, including the effects of DOR on miRNA splicing and maturation process, may lead to an alternative and reliable way for the DOR-miRNA–mediated neuroprotection against hypoxic/ischemic brain injury.

## Author Contributions

Y-MC searched the literature and prepared the manuscript. X-ZH and S-MW participated in the preparation of the manuscript. YX initiated the project, made the outline and revised the manuscript.

### Conflict of Interest

The authors declare that the research was conducted in the absence of any commercial or financial relationships that could be construed as a potential conflict of interest.

## Abbreviations

3′-UTRs: 3′ untranslated regions
AD: Alzheimer disease
BBB: Blood–brain barrier
ChIP: chromatin immunoprecipitation
DGCR8: DiGeorge syndrome critical region 8
DOR: δ-opioid receptors
GPD1L: glycerol-3-dehydrogenase-like 1
HIF-1: hypoxia inducible-factor 1
HRE: hypoxia-response-element
IR: Ischemia–reperfusion
IRAK1: interleukin-1 receptor-associated kinase 1
KOR: kappa-opioid receptor
MCAO: middle cerebral artery occlusion
miRNAs: microRNAs
MOR: mu-opioid receptor
mRNA: messenger RNA
OGD: oxygen–glucose deprivation
PD: Parkinson disease
pre-miRNAs: precursor miRNAs
pri-miRNAs: primary miRNAs
RISC: RNA-induced silencing complex
TLR4: Toll-like receptor 4
TRAF6: TNF receptor associated factor 6.
